# Extraction and Optimization of Potato Starch and Its Application as a Stabilizer in Yogurt Manufacturing

**DOI:** 10.3390/foods7020014

**Published:** 2018-01-29

**Authors:** Ammar B. Altemimi

**Affiliations:** Department of Food Science, College of Agriculture, University of Basrah, Basrah 61004, Iraq; ammaragr@siu.edu or ammar.ramddan@uobasrah.edu.iq

**Keywords:** yogurt, syneresis, microbial counts, gelatin, stabilizers, potato starch

## Abstract

Starch is increasingly used as a functional group in many industrial applications and foods due to its ability to work as a thickener. The experimental values of extracting starch from yellow skin potato indicate the processing conditions at 3000 rpm and 15 min as optimum for the highest yield of extracted starch. The effect of adding different concentrations of extracted starch under the optimized conditions was studied to determine the acidity, pH, syneresis, microbial counts, and sensory evaluation in stored yogurt manufactured at 5 °C for 15 days. The results showed that adding sufficient concentrations of starch (0.75%, 1%) could provide better results in terms of the minimum change in the total acidity, decrease in pH, reduction in syneresis, and preferable results for all sensory parameters. The results revealed that the total bacteria count of all yogurt samples increased throughout the storage time. However, adding different concentrations of optimized extracted starch had a significant effect, decreasing the microbial content compared with the control sample (Y_C_). In addition, the results indicated that coliform bacteria were not found during the storage time.

## 1. Introduction

Yogurt is a dairy product that has been known and widely consumed for a long time because it is beneficial for nutrition and has significant health effects [1]. Various types of yogurt are available on the market, such as liquid yogurt, sweetened, plain, flavored, frozen, and stirred yogurt. It is thought to prolong human life because of its protein content and minerals, in addition to being a good source of vitamin B [2,3]. Human consumption of yogurt has been linked with health benefits due to improved digestive function and a reduced risk of disease [4,5]. Scientists and researchers also pointed out the possibility of consuming yogurt instead of milk, especially for children and adults who suffer from lactose intolerance, because of its low lactose content [6].

Stabilizers are important ingredients in manufactured dairy products because of their capability to improve viscosity and sensory properties, and inhibit or decrease whey separation during storage, as well as enhance the ratio of total solids in manufactured dairy products [7]. Stabilizers have also been reported to show several secondary functional properties, but we need to assess their impact on physical, chemical, and sensory properties [8]. There are many sources of stabilizers. Some are synthetic (for example Carboxyl Methyl Cellulose); many of them have a plant origin, which is considered the cheapest and includes the most widely used ones such as corn starch, while a few, like gelatin, are of animal origin [9,10]. Gelatin is one of the most important stabilizers used in manufactured dairy products because it has a great effectiveness in increasing the viscosity and improving the qualities of dairy products [11]. However, the use of gelatin has decreased in recent years because of the cost as well as an increased demand for Halal and natural stabilizers and growing concern from consumers about using animal sources of gelatin [12].

Starch is increasingly used as a functional group either in industrial applications or food due to its ability to work as a thickener [13]. Starch is also widely used in yogurt manufacturing as a thickener to reduce defects, making the body and texture of manufactured yogurt appealing as well as reducing cracks in the surface of the curd milk [14,15]. Therefore, many plants were used to extract starch. For instance, Ammar et al. [16] suggested using Taro (*Colocassia esculenta*) because it is a good source of starch (70–80%), in addition to the ease of digestion and its positive effect on the properties of the final products. Potato is also an important ingredient for nutrition because it is a good source of starch, vitamins A and C, and minerals such as iron and potassium, in addition to different ratios of fibers [17].

*Ipomoea batatas* has been used as a major source of extracting starch in large quantities, especially in developed countries, where potato production accounts for 95% of total world food production [18]. The major objectives of this research are: (1) to extract and study the effect of extraction parameters X_1_ (centrifugal speed, rpm) ranging from (1000 to 3000) rpm and X_2_ (centrifugal time, min) ranging from (5 to 15) min on the yield of starch from yellow skin potato; (2) to determine the quality of yogurt stabilized with potato starch during storage for 15 days in a refrigerator.

## 2. Materials and Methods

Yellow skin potatoes for extraction of starch were obtained from the local market in Basrah city, Iraq. Fresh cow’s milk for making yogurt was obtained from the dairy farm of the College of Agriculture, University of Basrah, Basrah, Iraq. Gelatin and a freeze-dried starter culture of *Streptococcus thermophilus* and *Lactobacillus bulgaricus* were provided by the Department of Food Science, College of Agriculture, University of Basrah.

### 2.1. Extraction of Starch from Yellow Skin Potato

Six hundred grams of potatoes were washed thoroughly, peeled, sliced, and chopped into small chunks. The distilled water was added to the chopped potato and the extraction process was carried out through the use of a centrifuge at different speeds (1000, 2000, 4000) rpm for different periods of time (5, 10, 15 min). Thereafter, the centrifuged samples were filtered using Whatman no. 1 and the supernatant was neglected to obtain wet starch. The wet starch was dried at room temperature for 5 h, then crushed into a fine powder and stored in sealed containers for later use.

### 2.2. Preparation of Yogurt

The raw milk was filtered of impurities using clean gauze. Then, the extracted starch under the optimized condition was added at different concentrations, as shown in Table 1. Gelatin (0.6%) was used as a standard sample to make yogurt. Then, the temperature of milk was gradually increased to 90 °C for 30 min with constant stirring to make sure the extracted starch dissolved. Pasteurized cow’s milk was rapidly cooled to 43 °C for the purpose of adding 3% (*w*/*v*) of starter culture and stirring for 4 min. Thereafter, sterilized plastic containers with a tight seal were completely filled with milk and transferred to an incubator, where they were kept at 42 °C for 4 h until the completion of coagulation. Manufactured yogurt was stored in a refrigerator at 5 °C [19].

### 2.3. Analysis of Yogurt

The pH, acidity, and syneresis of manufactured yogurt were measured in triplicate to avoid error during the 15-day storage at 5 °C.

### 2.4. Acidity of Yogurt

The acidity value was calculated based on the method described by Onwuka [20]. The acidity value was estimated as the amount of 0.1 N NaOH solution (mL) used to neutralize 10 g of yogurt samples, using phenolphthalein as an indicator to achieve a pink color.

### 2.5. pH of Yogurt

The pH was measured with an electronic digital-type pH meter (WTW series pH-720). Firstly, the electrodes of pH meter were adjusted and calibrated at room temperature using buffer solutions of pH 4 and 7. Then, electrodes of pH meter were immersed in a beaker containing 5 g of yogurt and readings were recorded directly [21].

### 2.6. Syneresis of Yogurt

The degree of syneresis was determined as free whey according to the method mentioned by Al-Kadamany et al. [22]. Ten-gram samples of manufactured yogurt were weighed and directly placed on a funnel containing Whatman no. 1 filter paper. The syneresis was assessed according to the following equation after 10 min of drainage under vacuum conditions:

Free whey (g/100 g) = (W_b_ − W_a_/W_b_) × 100, (1)

where W_b_: weight of yogurt before drainage, W_a_: weight of yogurt after drainage.

### 2.7. Microbiological Analysis of Yogurt

The total bacteria count can be determined by making a serial dilution to 10 of one gram of each sample of yogurt. Thereafter, 0.1 mL of each sample of yogurt was placed on nutrient agar plates and incubated at 35 °C for 48 h. The same procedure was used for counting coliform bacteria, except that nutrient agar was replaced with MacConkey agar, and all petri dishes were incubated at 37 °C [23].

### 2.8. Sensory Evaluation

The overall acceptability of extracted potato starch in yogurt manufacturing was carried out by a panel of 20 trained panelists from the staff of the Food Science Department, College of Agriculture, University of Basrah according to the method described by Sameen et al. [12]. Appearance, body and texture, flavor, and acidity were assessed for sensory evaluation of manufactured yogurt. The sensory evaluation was done on day 1, day 5, day 10, and day 15 of storage.

### 2.9. Experimental Design and Data Analysis

Two independent variables and three coded levels (−1, 0 and +1) were used as effective factors: X_1_ (centrifugal speed, rpm) ranged from (1000 to 3000) rpm and X_2_ (centrifugal time, min) ranged from (5 to 15) min, while the dependent variable (response variable) was the yield of extracted potato starch. The optimal extraction condition was achieved using a central composite design. The following second-order polynomial model was used to describe the relationship between the two independent variables and the response variable:

Y_i_ = b_0_ + b_1_X_1_ + b_2_X_2_ + b_12_X_1_X_2_ + b_11_X_1_^2^ + b_22_X_2_^2^, (2)

where Y_i_ is the predicted response; b_0_ is an intercept; b_1_ and b_2_ are the estimated coefficients of centrifugal speed (X_1_) and time (X_2_), respectively; b_11_ and b_22_ are quadratic effects; and b_12_ is interaction effect of independent variables. The experimental results were analyzed using the statistical software Design Expert 10.6 (State-Ease Inc., Minneapolis, MN, USA).

## 3. Results and Discussion

Table 2 shows the experimental values of extracting starch from yellow skin potatoes, indicating the processing conditions at 3000 rpm and 15 min as optimum for the highest yield of extracted starch. From the analysis of variance shown in Table 3, the model was highly significant (*p* < 0.05), which indicated that the models used to fit response variables were sufficient to display the relationship between the yield of starch and the independent variables. The “Lack of Fit *F*-value” of 2.06 implies the lack of fit is not significant relative to the pure error. There is a 24.32% chance that a “Lack of Fit *F*-value” this large could occur due to noise. Non-significant lack of fit is recommended for an adequate model. Both linear and square of X_1_ (centrifugal speed, rpm) also showed a *p*-value lower than 0.05. Furthermore, the linear and square of X_2_ (centrifugal time, min) also showed a *p*-value lower than 0.05. The interaction between X_1_ (centrifugal speed, rpm) and X_2_ (centrifugal time, min) gave *p* < 0.05, which is considered significant. The *R^2^* of the models for potato starch yield (%) was 0.946.

Three-dimensional (3D) surface plots were assigned in order to study and determine the optimum conditions for independent and dependent variables [24]. The equation in terms of coded factors can be used to make predictions about the response for given levels of each factor. By default, the high levels of the factors are coded as +1 and the low levels of the factors are coded as −1. The coded equation is useful for identifying the relative impact of the factors by comparing the factor coefficients. The quadratic polynomial model of coded factors is shown below:

Potato Starch Yield % = +8.50 + 2.44×X_1_ + 1.56×X_2_ + 1.50×X_1_X_2_ + 1.38×X_1_^2^ + 0.38×X_2_^2^. (3)

**Figure 1:**
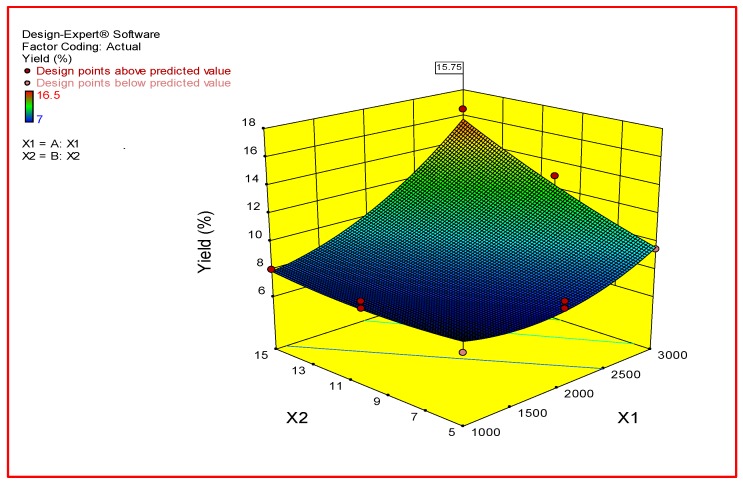
Response surface plot showing the effect of centrifugal speed and centrifugal time on the potato starch yield (%).

As shown in Figure 1, the effect of the variables and their interaction on predicted potato starch yield (%) was investigated. It showed that as X_1_ (centrifugal speed) and X_2_ (centrifugal time) increased, the potato starch yield increased. The optimum centrifugal speed and centrifugal time for maximum potato starch yield were 3000 rpm and 15 min, respectively.

### 3.1. Total Acidity in Yogurt Samples

Table 4 shows the effect of adding the extracted starch from the potatoes under the optimized condition on the total acidity in yogurt stored at 5 °C for 15 days. The results of the statistical analysis showed that Yp_1_ and Yp_2_ exhibited a maximum change in total acidity over storage and were significantly (*p* < 0.05) higher than Y_G_, Yp_3_, and Yp_4_, which was not significant (*p* > 0.05) compared to Y_C_. The mean values of total acidity were 1.35 ± 0.56, 1.33 ± 0.56, and 1.4175 ± 0.54 for Yp_1_, Yp_2_, and Y_C_, respectively. This result was in agreement with those of Andic et al. [25] and Anwer et al. [26], who reported a significant relationship between the gradual increase in acidity of yogurt during the storage and the amount of lactic acid produced.

In spite of Yp_3_ and Yp_4_ showing a slight increase in the total acidity of manufactured yogurt during storage at 5 °C for 15 days, the statistical analysis showed that there were no significant differences (*p* > 0.05) between Yp_3_, Yp_4_, and Y_G_. This obtained result was in agreement with Kumar and Mishra [27], who found that adding sufficient concentrations of starch could effectively reduce the amount of water, thus making it difficult for bacteria to metabolize lactose sugar and thereby reducing the amount of lactic acid produced.

### 3.2. pH of Yogurt Samples

The results in Table 5 show that adding the starch extracted from yellow potato had a significant effect on the mean value of pH of yogurt samples. The statistical analysis implied that Yp_1_ and Yp_2_ presented the maximum decrease in pH values and were significantly (*p* < 0.05) less than Y_G_, Yp_3_, and Yp_4_, which was not significant (*p* > 0.05) compared to Y_C_. The mean values of total acidity were 4.16 ± 0.45, 4.205 ± 0.39, and 4.10 ± 0.47 for Yp_1_, Yp_2_, and Y_C_, respectively. These findings were similar to those reported by Seelee et al. [28] and Hassan et al. [29], who declared that the pH value of yogurt decreased mainly because of the lactic acid produced during storage.

Furthermore, the results revealed that Yp_3_ and Yp_4_ displayed a negligible decrease in the pH of manufactured yogurt during storage at 5 °C for 15 days. The Yp_3_ and Yp_4_ treatments had more capability to resist pH changes due to their ability to prevent lactose conversion [30]. The statistical analysis showed that there was no significant difference (*p* > 0.05) between Yp_3_, Yp_4_, and Y_G_.

### 3.3. Syneresis of Yogurt Samples

As shown in Table 6, adding different concentrations of extracted starch had highly significant results, decreasing syneresis in manufacturing yogurt during storage at 5 °C for 15 days. This study indicated that Yp_1_ and Yp_2_ exhibited the minimum reduction in syneresis with the passage of time and were significantly (*p* < 0.05) higher than Y_G_, Yp_3_, and Yp_4_, which was not significant (*p* > 0.05) compared to Y_C_. The mean values of syneresis were 4.32 ± 1.40, 4.38 ± 1.39, and 4.45 ± 1.39 for Yp_1_, Yp_2_, and Y_C_, respectively. This result was in accordance with Isleten et al. [31] and Guven et al. [32], who observed that the lowest values of syneresis were obtained during storage compared to the first day of production. 

In contrast, both Yp_3_ and Yp_4_ treatments displayed preferable results in terms of the reduction in syneresis values during storage at 5 °C for 15 days. This significant reduction can be ascribed to the ability of a high concentration of starch to increase the concentration of an adsorbing polymer. Previous results of Hasan et al. [33] were in agreement with this present investigation. Moreover, the statistical analysis emphasized that there were no significant differences (*p* > 0.05) between Yp_3_, Yp_4_, and Y_G_.

### 3.4. Microbiological Analysis of Yogurt

**Figure 2:**
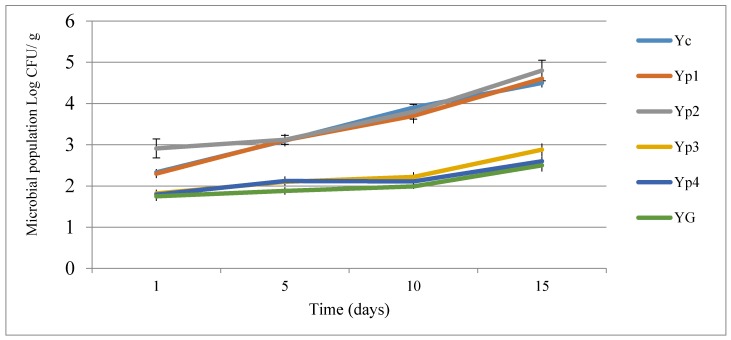
Effect of adding optimized extracted starch on the microbial population of yogurt during storage.

The data regarding microbial population changes of all yogurt samples are given in Figure 2. The results revealed that the total bacteria count in all yogurt samples increased throughout the storage time. Adding different concentrations of optimized extracted starch had a significant effect, decreasing the microbial content compared with the control sample (Y_C_). This study indicated that Yp_1_ and Yp_2_ exhibited the minimum reduction in microbial content with the passage of time and were significantly (*p* < 0.05) higher than Y_G_, Yp_3_, and Yp_4_, which was not significant (*p* > 0.05) compared to Y_C_. The mean values of the total bacteria count (log10 CFU/mL) during storage at 5 °C for 15 days were 4.5 ± 0.23, 4.6 ± 0.11, and 4.8 ± 0.18 for Yp_1_, Yp_2_, and Y_C_, respectively. However, both Yp_3_ and Yp_4_ treatment displayed preferable results in terms of the reduction in microbial content during storage at 5 °C for 15 days. Moreover, the statistical analysis emphasized that there were no significant differences (*p* > 0.05) between Yp_3_, Yp_4_, and Y_G_. This present investigation was not in agreement with previous results of Hasan et al. [33] and Dave et al. [34], who confirmed that there were no significant effects due to different concentrations of stabilizers.

In addition, the results also indicated that coliform bacteria were not found during storage. This result was in accordance with Hasan et al. [33] and Ganesh [35], who confirmed the absence of coliform bacteria because of good storage requirements and avoiding contamination.

### 3.5. Sensory Evaluation

**Figure 3:**
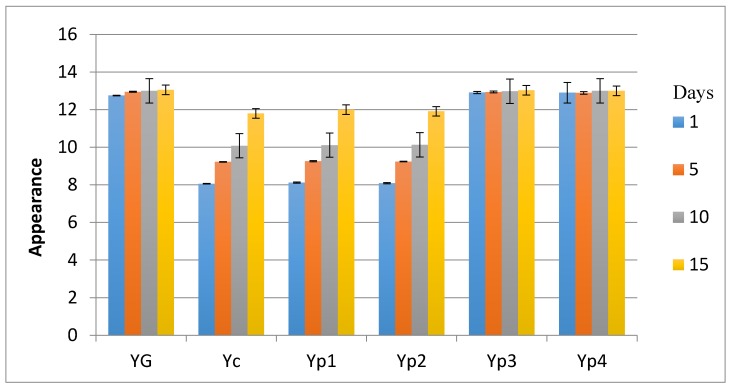
Mean values of yogurt’s appearance during storage.

**Figure 4:**
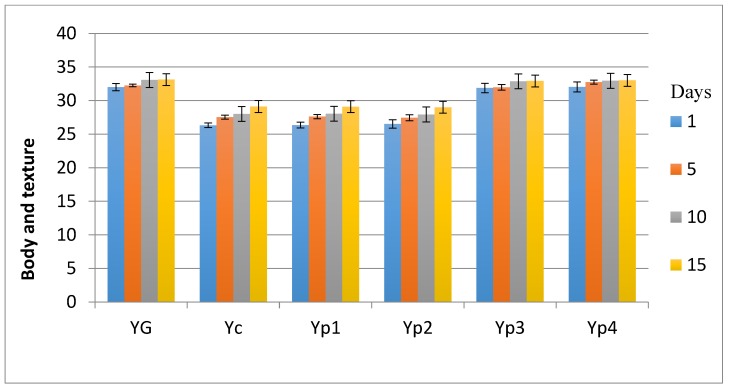
Mean values of yogurt’s body and texture during storage.

**Figure 5:**
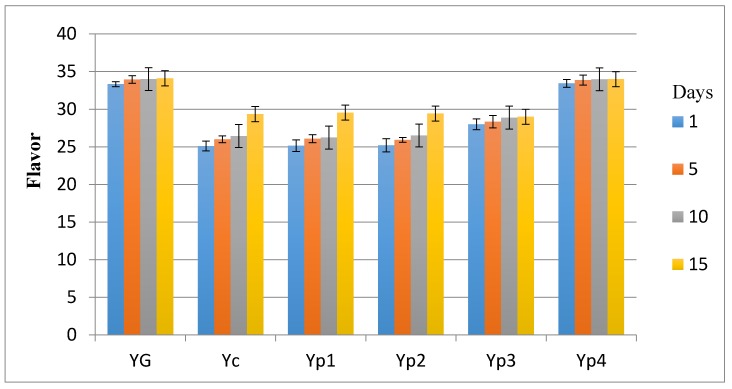
Mean values of yogurt’s flavor during storage.

**Figure 6:**
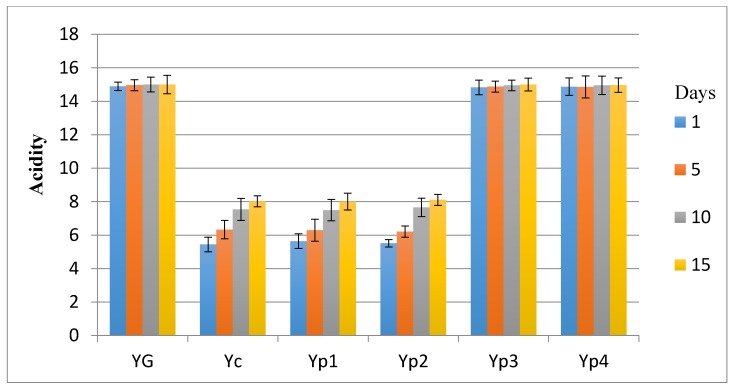
Mean values of yogurt’s acidity during storage.

The results in the sensory investigation included appearance, texture, flavor, and acidity, as shown in Figure 3, Figure 4, Figure 5 and Figure 6. The statistical analysis emphasized that there was no significant difference (*p* > 0.05) between Yp_3_, Yp_4_, and Y_G_ for all sensory parameters. This study also indicated that Yp_1_ and Yp_2_ had the lowest scores in terms of all sensory parameters with the passage of time and were significantly (*p* < 0.05) lower than Y_G_, Yp_3_, and Yp_4_; however, it was not significant (*p* > 0.05) compared to Y_C_. This finding was similar to those reported by Malik et al. [7], who confirmed that yogurt samples remained satisfactory during storage at 5 °C for 15 days for all sensory parameters due to the different concentration of starch extracted from *Trapa bispinosa*. In contrast, this present study was not in agreement with Sameen et al. [12], who said that there was no statistical difference between adding a different concentration of starch in manufacturing yogurt and the control sample for all sensory parameters.

## 4. Conclusions

Stabilizers are important ingredients in manufactured dairy products because of their ability to improve viscosity and sensory properties, and to decrease whey separation during storage. The results showed that adding sufficient concentrations of extracted starch (0.75%, 1%) gave better results for the total acidity, pH, syneresis, and sensory evaluation. Both Yp_3_ and Yp_4_ treatments displayed preferable results in terms of reduction in microbial content during storage at 5 °C for 15 days. Furthermore, yogurt bacterial counts were also significantly reduced using different concentrations of extracted starch throughout storage.

## Figures and Tables

**Table 1:** The work plan within the optimized condition.

Yogurt Treatment	Extracted Potato Starch % (*v*/*w*)	Gelatin % (*v*/*w*)
Y_G_	-	0.6
Y_C_	0	-
Yp_1_	0.25	-
Yp_2_	0.5	-
Yp_3_	0.75	-
Yp_4_	1	-

Where Y_G_ = standard (0.6% gelatin), Yc = control yogurt (without any stabilizer), Yp_1_ = 0.25% extracted potato starch, Yp_2_ = 0.5% extracted potato starch, Yp_3_ = 0.75% extracted potato starch, Yp_4_ = 1% extracted potato starch.

**Table 2:** The experimental values using central composite design.

Run	X_1_ (rpm)	X_2_ (Min)	Potato Starch Yield %
1	3000	10	11
2	1000	15	8
3	2000	5	8
4	1000	10	7.5
5	3000	5	9.5
6	3000	15	16.5
7	2000	15	10
8	1000	5	7
9	1000	10	8
10	2000	5	7.5
11	2000	15	10
12	3000	10	13

X_1_: centrifugal speed; X_2_: centrifugal time.

**Table 3:** The analysis of variance of the fitted quadratic model for potato starch yield (%).

Source	Degree of Freedom	Sum of Square	Mean Square	*F*-Value	*p*-Value
Model	5	80.10	16.02	21.07	0.001
X_1_	1	47.53	47.53	62.51	0.0002
X_2_	1	19.53	19.53	25.68	0.0023
X_1_X_2_	1	9	9	11.84	0.013
X_1_^2^	1	3.78	3.78	4.97	0.067
X_2_^2^	1	0.28	0.28	0.37	0.565
Lack of fit	2	2.31	1.16	2.06	0.2432

**Table 4:** Mean values of total acidity in yogurt manufacturing at 5 °C for 15 days.

Storage Period (Days)	Yogurt Treatments *					
	Y_C_	Yp_1_	Yp_2_	Yp_3_	Yp_4_	Y_G_
1	0.75	0.75	0.74	0.49	0.53	0.55
5	1.22	0.98	0.95	0.42	0.44	0.41
10	1.75	1.78	1.75	0.51	0.52	0.48
15	1.95	1.89	1.88	0.61	0.59	0.53
Means	1.41 ± 0.54 ^a^	1.35 ± 0.56 ^a^	1.33 ± 0.56 ^a^	0.50 ± 0.07 ^b^	0.52 ± 0.06 ^b^	0.49 ± 0.06 ^b^

* Means with the same superscript letter are not significantly different.

**Table 5:** Mean values of pH in yogurt manufacturing at 5 °C for 15 days.

Storage Period (Days)	Yogurt Treatments *					
	Y_C_	Yp_1_	Yp_2_	Yp_3_	Yp_4_	Y_G_
1	4.74	4.75	4.71	4.75	4.74	4.74
5	4.16	4.21	4.25	4.73	4.75	4.72
10	3.89	4.03	4.11	4.69	4.72	4.70
15	3.64	3.67	3.75	4.61	4.63	4.59
Means	4.10 ± 0.47 ^a^	4.16 ± 0.45 ^a^	4.20 ± 0.39 ^a^	4.69 ± 0.06 ^b^	4.71 ± 0.05 ^b^	4.68 ± 0.06 ^b^

* Means with the same superscript letter are not significantly different.

**Table 6:** Mean values of syneresis (g/100 g) in yogurt manufacturing.

Storage Period (Days)	Yogurt Treatments *					
	Y_C_	Yp_1_	Yp_2_	Yp_3_	Yp_4_	Y_G_
1	6.15	5.98	6.08	2.21	2.19	2.18
5	4.85	4.91	4.81	2.15	2.01	2.07
10	3.92	3.65	3.83	1.87	1.86	1.91
15	2.88	2.77	2.81	1.79	1.77	1.75
Means	4.45 ± 1.39 ^a^	4.32 ± 1.40 ^a^	4.38 ± 1.39 ^a^	2.0 ± 0.20 ^b^	1.95 ± 0.18 ^b^	1.97 ± 0.18 ^b^

* Means with the same superscript letter are not significantly different.
